# Pharmacology Knowledge Among Nurses Working in Nursing Homes in Norway: A Cross-Sectional Study

**DOI:** 10.1177/23779608241303482

**Published:** 2024-12-05

**Authors:** Siv Eriksen Taasen, Fred-Ivan Kvam, Kjersti Marie Blytt, El Houcine Messaoudi

**Affiliations:** 1 Faculty of Health and Social Sciences, Department of health and Caring Sciences, 1657 Western Norway University of Applied Sciences, Bergen, Norway

**Keywords:** knowledge of pharmacology, medication management, nurses, nursing homes, cross sectional

## Abstract

**Introduction:**

The administration of drugs is an important part of nurses’ professional practice. A basic knowledge of pharmacological principles is required to make accurate decisions about drug management and conduct patient medication education. However, several studies have suggested that nurses have inadequate knowledge of pharmacology and medication management.

**Objective:**

To explore the level of knowledge in pharmacology and medication management among nurses working in nursing homes (NHs).

**Methods:**

We conducted a cross-sectional study at 24 NHs in Norway. The nurses took a multiple-choice test in pharmacology with 35 questions at their workplace. Each question had four alternative answers with one answer being correct. One point was awarded for a correct answer and zero for a wrong answer. The test included categories in general pharmacology, clinical pharmacology, and medication management.

**Results:**

In total, 145 nurses completed the questionnaire. In the range of 0–35 correct responses, the mean score for the sum of all questions was 22.5, which equates to 66% of responses being correct. Linear multiple regression showed no association regarding gender, age distribution among the nurses, years since completing basic education in nursing, years of work experience in NHs, postgraduate education, and knowledge of pharmacology and medication management. Knowledge was better in the category medication management (*p* < 0.001) than in the category's general pharmacology and clinical pharmacology. When evaluating specific test questions, 38.9% of the nurses answered a question on opioids correct, 64.1% a question on anticoagulants correct, 33.5% knew the prerequisites for generic substitution, and 45.5% did not know the difference between agonists and antagonists.

**Conclusion:**

Our results are in line with previous findings. The participating nurses working in the NHs have insufficient knowledge in pharmacology and medication management. This may cause harm and undermine patient safety.

## Introduction

Nurses have a central role in medication management (Huisman et al., 2020). This involves administering medication safely and efficiently, assessing and monitoring effects, and providing patients with knowledge and skills regarding their medication (Baker & Naphthine, 1994; Huisman et al., 2020; Manias & Bullock, 2002). This highly desired competence requires practical and theoretical skills, including medication calculation and knowledge of pharmacology (Karttunen et al., 2020). However, several studies indicate that nurses have low levels of competence in pharmacology (Boggs et al., 1988; Brady et al., 2009; Gigi & Honey, 2017; Meechan et al., 2011; Ndosi & Newell, 2009; Simonsen et al., 2011). Elderly patients exhibit an age-dependent decline in physiological functions and changes in the composition of the organism leading to altered pharmacokinetic and pharmacodynamic mechanisms. Among the pharmacokinetic and pharmacodynamic changes in the elderly is the reduction in renal drug elimination, resulting in increased drug serum levels. Age-related decrease in liver metabolic clearance of drugs, and a progressive age-dependent decline in the homeostatic mechanisms (Tan et al., 2015). Furthermore, the body water volume diminishes while the body fat increases leading to alteration in hydrophilic and lipophilic drug distribution volumes (Turnheim, 2004). Knowledge in these age-related changes is necessary for nurses in order to give an adequate care for elderly patients. Comorbidity with subsequent polypharmacy is common among elderly patients and is associated with a greater risk of drug interactions, adverse reactions, reduced functional capacity, and hospitalization (Kurczewska-Michalak et al., 2021; Maher et al., 2014).

Medication noncompliance is a frequent consequence of polypharmacy. The consequences of noncompliance are often poor health outcomes, increased comorbidities, and death (Chisholm-Burns & Spivey, 2012). A higher number of drugs taken by the patient are associated with a higher number of adverse drug reactions (Viktil et al., 2007). Several studies have shown that residents in nursing homes (NHs) use approximately eight drugs (Halvorsen et al., 2010; Onder et al., 2012; Soraas et al., 2014). In a Norwegian setting, for safety reasons, physicians should avoid prescribing drugs figuring in the Norwegian General Practice (NORGEP) criteria list to elderly patients as the list containing explicit criteria for pharmacologically inappropriate prescriptions (Rognstad et al., 2009). However, results indicate that approximately 30% of the elderly population are prescribed medication from the NORGEP list (Nyborg et al., 2012). There is no reason to believe that this does not also apply to other countries. In light of this, knowledge regarding drug effect, side effects, and drug interactions is of key importance since nurses are responsible for these vulnerable patients on an everyday basis.

Simonsen et al. (2014) conducted a comparative study and found that Norwegian nurses have inadequate medication knowledge. Furthermore, one out of four nurses showed a high risk of error in drug handling because of lack of knowledge regarding side effects, interactions, and administration forms. Their results also indicate that nurses’ knowledge of pharmacology increased during their first-year working as a nurse. However, there was no further improvement after they had acquired more job experience (Simonsen et al., 2014).

Previous studies have investigated specific topics such as medication error in nursing practice (Brady et al., 2009; Furjanic et al., 2016), the pharmacology of pain management (Furjanic et al., 2016) and pharmacology knowledge in resuscitation medication (Qedan et al., 2022). However, no prior multicenter study has examined knowledge in pharmacology and medication management among nurses working in NHs. Consequently, the aim of this study is to assess knowledge of pharmacology and medication management and different levels of knowledge when it comes to general pharmacology, clinical pharmacology, and drug management, among nurses working in NHs in Norway, by means of a multiple-choice questionnaire. We also aim to explore if nurses’ work experience in NHs, number of years since completing bachelor's degree and postgraduate education influence their competence in pharmacology and medication management. Furthermore, we assess nurses’ knowledge of specific clinical topics referred to as high-risk medication, especially opioids and anticoagulants, two drug groups commonly used by elderly patients (Davies, 2017; Shehab et al., 2016). Knowledge in generic substitution and the concept of agonist/antagonist are also assessed.

## Methods

### Design, Participants, and Setting

We sent an invitation to all 32 NHs in a large municipality in Norway. Twenty-four NHs chose to participate. Nursing home managers distributed our email with information about the study and an invitation to participate, as well as a link to the questionnaire through an online survey platform (Survey Xact 8.2) to about 400 registered nurses. Through e-mail and staff meetings in the NH, the informants were encouraged to answer the questionnaire at their workplace. The inclusion criteria for participation in the study were working as a registered nurse in the actual NHs. There was no time limitation for responding to the survey after the questionnaire was opened.

The data were collected between September 15, 2021, and October 15, 2021. During this period, the managers of the NHs received weekly emails with a reminder about the survey and a request to recruit more nurses.

### Questionnaire Development and Validation

The municipal NHs agency was included in the project group and participated in the design and approval of the questionnaire. The questionnaire's content was examined for face validity by two medical doctors and one lead pharmacist working in NHs. In addition, the questionnaire was tested by two eligible nonparticipating nurses, and we adjusted the questionnaire on the basis of their feedback. The questionnaire allows us to directly test nurses’ knowledge of topics they deal with on a daily basis. We also asked in the questionnaire about demographic characteristics, gender, age, work experience in NHs, and number of years since completing bachelor's degree and postgraduate education.

### Questionnaire Design and Content

We developed a multiple-choice questionnaire (pharma-test 1). Each question had four alternative answers, but only one correct answer. The questionnaire was organized into three categories: (1) general pharmacology: seven questions (including generic substitution, interactions, therapeutic margin, and side effects); (2) clinical pharmacology: 22 questions (including drugs used for circulatory, respiratory, gastrointestinal and musculoskeletal disorders, analgesics, hypnotics, antibiotics, and drugs used in palliative care and emergency situations), and (3) medication management: six questions (including tablet crushing and oral and parenteral drug administration). The test questions were developed on the basis of topics in pharmacology and medication management that are necessary on a daily basis for practice in NHs.

The Pharma-test 1 questionnaire includes nine questions that reflect aspects of pharmacology knowledge considered to be of high clinical importance for nurses practicing in NHs. These nine questions address the following topics: (1) tablet crushing; (2) generic substitution, (3) use of high-risk medications such as opioids (morphine), anticoagulants (warfarin), and epinephrine/adrenaline (anaphylaxis), (4) diuretics (furosemide) against acute pulmonary edema, and (5) medication for symptom relief in final phase of life. In addition, we comment upon the specific questions asked in the questionnaire on important topics related to safe medication such as the use of opioids and anticoagulants, understanding the difference between agonists and antagonists, and the use of generic substitution.

### Ethical Consideration

The Norwegian Agency for Shared Services in Education and Research approved the study (No. 901714). The ethics committee at the Western Norway University of Applied Sciences also approved the study. All participants received written information about the study. By continuing to answer the questionnaire, they gave their written consent to participate. They typed in a password they could use if they would like to withdraw their consent later. None of the participants contacted us to withdraw their consent.

### Statistical Analyses

Descriptive statistics were calculated for all relevant measurements. We calculated the percentage of the participant nurses giving correct answers to the questions in our test. Linear multiple regression was used to explore how age, years since nursing training was completed, years of work experience as a nurse in a NH and postgraduate education correlated with knowledge of pharmacology and medication management. We created dummy variables used as reference categories for (1) ages of 26–35, (2) more than 15 years’ experience following basic education, and (3) less than 5 years’ work experience in NHs and (4) having postgraduate education (see Table 1). The questionnaire was organized into three categories (general pharmacology, clinical pharmacology, and medication management). We calculated sum score variables for each category, where we divided the sum score in the category by the number of questions in the category. This yielded comparable percentages for each category. We used paired *t*-tests to compare the percentage of correct answers in the three categories. The data were analyzed using IBM SPSS version 29. We regarded *p*-values of less than 5% as significant.

**Table 1.** Demographic Data: Number of Participants, Gender, Age Distribution Among the Nurses, Years Since Completing Basic Education in Nursing, Years of Work Experience in Nursing Homes, and Postgraduate Education.

Characteristics		Number	%
Number of participants		145	
Age distribution among the nurses	20–25 years	6	4.1
	26–35 years	49	33.8
	36–45 years	39	26.9
	46–55 years	33	22.8
	56–65 years	18	12.4
Gender	Male	6	6.2
	Female	139	93.8
Number of years since completing bachelor's degree (in nursing)	1–5 years	34	22.1
	6–10 years	32	19.3
	11–15 years	28	35.2
	More than 16 years	51	23.4
Experience working as a nurse in a nursing home	1–5 years	55	37.9
	6–10 years	34	23.4
	11–15 years	30	20.7
	More than 16 years	26	17.9
Postgraduate education*	30 credits or more	52	35.8

* Formal education of more than 30 credits (geriatric nursing, mental health, palliative care, administration etc.).

## Results

**Figure 1.**
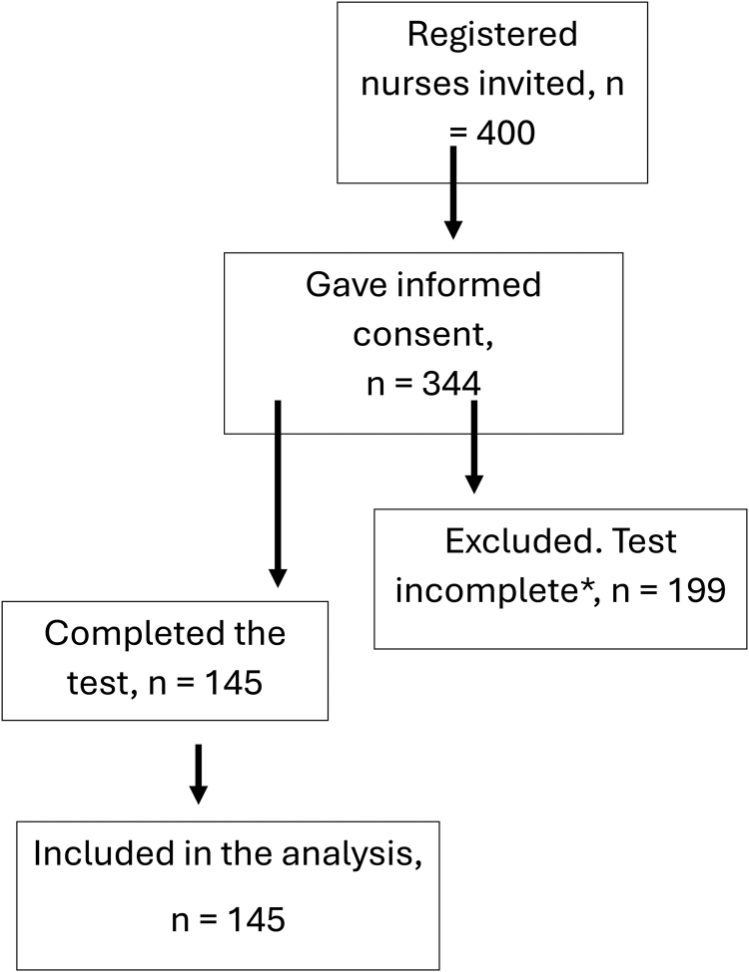
Number of invited nurses, exclusion process and the final number of included participants. * included in the analyses of answers to individual questions.

From the 24 participating NHs, 344 nurses gave informed consent and started the test; 199 were excluded because they did not answer all of the questions (see Figure 1). However, when analyzing each individual question, the actual number of participants who had answered the question was used. The average length of time spent taking the test was 41 min. The final sample included 145 participants. Of these, 93.8% were female (*n* = 139). Age distribution among nurses showed that 64.8% were under 46 years; 37.9% of participants had less than 6 years working experience from NHs and approximately one-third had postgraduate education. See Table 1 for an overview of number of participants, gender, age distribution among the nurses, years since completing basic education in nursing, and years of work experience in NHs and postgraduate education.

In the range of 0–35 correct responses, the lowest score was 14 and the highest score was 32. The mean score for the sum of all questions was 22.5, which equates to 66% of answers being correct (median = 23; 66%). Based on Biddle (1993), we decided to categorize the results such that a correct response rate of 70% equated to acceptable competence in pharmacology and medication management; 35.1% of the participants scored 70% or better on the test. See Table 2 for the percentage of nurses’ giving correct response to the questions in our test.

**Table 2.** Number and Percentage of Nurses Giving Correct Answers to the 35-Item Pharmacology and Medication Management Test.

Correct answers (%)	Number of nurses	Percentage of nurses
0–49	19	13.1
50–69	75	51.7
70–85	44	30.3
86–100	7	4.8
SUM	145	100.0

The linear multiple regression showed no association regarding gender, age distribution among the nurses, years since completing basic education in nursing, years of work experience in NHs, postgraduate education and knowledge of pharmacology and medication management (total sum score as dependent variable). See Table 3 for the full regression analyses.

**Table 3.** Multiple Linear Regression Exploring the Determinants of Knowledge in Pharmacology and Medication Management.

	B	SE	*Β*	T	*P*
Gender	−0.612	1.421	−0.038	−0.431	0.668
Age distribution (20–25)	0.318	1.843	0.016	0.174	0.863
Age distribution (36–45)	0.336	1.010	0.038	0.333	0.740
Age distribution (46–55)	−0.105	1,223	−0.011	−0.086	0.932
Age distribution (56–65)	0.454	1.458	0.038	0.312	0.756
Years since basic education (1–5)	1.604	1.548	0.173	1.036	0.302
Years since basic education (6–10)	0.560	1.341	0.059	0.418	0.677
Years since basic education (11–15)	0.053	1.191	0.005	0.045	0.965
Years as employed in NHs (6–10)	0.581	1.190	0.063	0.488	0.626
Years as employed in NHs (11–15)	0.898	1.397	0.093	0.643	0.551
Years as employed in NHs >16	2.055	0.839	0.103	1.110	0.269
Postgraduate education	0.932	0.839	0.103	1.110	0.269

B, nonstandardized coefficients; SE, standard errors; β, standardized coefficients; T, *T*-values, *P*, *p* values; NH, nursing home. For the age dummy variable, ages of 26–35 is used as reference category. For the years following nursing basic education dummy variable, more than 15 years’ experience is used as reference category. For the employment dummy variable, less than 5 years of employment is used as reference category.

In the category investigating knowledge of general pharmacology (seven questions), the results revealed a mean score of 0.59 (SD 0.20). In the category investigating knowledge of clinical pharmacology (22 questions), the results yielded a mean score of 0.63 (SD 0.12). Finally, in the category investigating knowledge of medication management, the results yielded a mean score of 0.75 (SD 0.17). Furthermore, we conducted paired sample *t*-tests between the three categories of questions. As shown in Table 4, there were significant differences in correct responses between the three categories of questions: at the 5% level between general pharmacology and clinical pharmacology, and at the 1% level between general pharmacology and medication management and between clinical pharmacology and medication management.

**Table 4.** Comparison of Mean Score on Each Question Divided into the Category's General Pharmacology, Clinical Pharmacology, and Medication Management.

Categories	Mean score (SD)	p* C_1_ vs C_2_	p* C_2_ vs C_3_	p* C_1_ vs C_3_
C_1_ General pharmacology (7 questions)	0.59 (0.20)	0.013		
C_2_ Clinical pharmacology (22 questions)	0.63 (0.12)		<0.001	
C_3_ Medication management (6 questions)	0.75 (0.17)			<0.001

p* < 0.05 significant by paired *t*-test.

Because inappropriate drug management and use of high-risk drugs can be harmful (Huang et al., 2021), we set the cut score to eight of nine right answers when evaluating the nurses’ knowledge of these medications. Regarding medications of specific importance for nurses in NHs to have knowledge about, only 18.6% of the participant nurses had eight or more out of nine answers correct. (Result not shown in table.) We assessed nurses’ knowledge of opioids and anticoagulants. The results show that 38.9% and 64.1% had right answers respectively (see Table 5).

**Table 5.** Examples of Questions from the Pharmacology Test Included the Given Alternative for Answers.

Questions from the pharmacology test	Number of nurses who answered the question*	Percent of nurses with correct answer
A patient is using an opioid (oxycodone) as treatment for strong pain. It has been decided that the treatment should change from oral to parenteral administration. This implicates that the patient will get: Alternative answers** (a) Higher dose. (b) Same dose. *(c) Lower dose*. d) Another opioid.	198	38.9
Marevan (warfarin) is used to prevent blood clots. Choose right statement about Marevan: Alternative answers: (a) Alcohol inhibits the effect of Marevan. (b) *Foods containing vitamin K lower INR, thus reducing the effect of warfarin*. (c) Marevan must be given as injection. (d) Marevan increases the formation of vitamin K-dependent coagulation factors.	170	64.1
You are about to hand out medication to a patient in nursing home, when you discover that you are running short for a drug figuring on the patient's drug list. What are the prerequisites for using generic drugs instead? Alternative answers: (*a) The state's pharmaceuticals agency has approved drugs as interchangeable*. (b) The drugs contain the same active ingredient in equal dose and concentration. (c) The drugs have the same pharmaceutical form. (d) The drugs are listed on the same ATC code in the Norwegian Pharmaceutical Product Compendium (Felleskatalogen/ The joint catalogue for medication).	198	33.5
Which statement about agonists and antagonists is correct? Alternative answers: (a) An antagonist is a drug that stimulates a receptor. (b) An agonist is a drug that blocks a receptor. *(c) A partial agonist is a drug that only provides a partial stimulation of a receptor.* (d) A drug that enhances the effect of an agonist is called a partial antagonist.	145	45.5

*The number of nurses who answered the actual question. The number might be higher than the number used for regression analyses.

**The correct answer is in *italics.*

For questions regarding knowledge in generic substitution 33.5% answered correct. On question exploring the conception of agonist/antagonist 45.5% gave correct answers (see Table 5).

## Discussion

The results of the present study show that only 35.1% of the participating nurses had correct answers to 70% or more of the questions. Nurses need adequate knowledge in pharmacology and medication management to be able to observe the effect of the treatment, any side effects of the treatment and be able to inform the patient about how drugs work (King, 2004).

In the different categories, the correct response percentages were 59% (general pharmacology), 63% (clinical pharmacology), and 75% (medication management). This indicates that the nurses included in the present study demonstrated an inadequate knowledge of pharmacology and medication management. The pharma-1 test shows significantly fewer correct answers in general pharmacology compared to clinical pharmacology and medication management. Furthermore, the results revealed a significantly lower level of knowledge of clinical pharmacology compared to medication management. These findings might be explained by a possible lack in clinical experience among nurses. In our study, 37.9% of the nurses had between one and five years’ work experience in NHs. Our results are supported by earlier studies suggesting that inadequate knowledge in pharmacology is more common among newly educated nurses (Dilles et al., 2011) since they may struggle to contextualize pharmacological knowledge in practice (Brady et al., 2009; Dilles et al., 2011). Improving knowledge in general and clinical pharmacology requires appropriation of theoretical knowledge through reading pharmacology textbooks and research articles. Earlier studies however show that many nurses still relay on personal experience and intuition and not on implementing valid and applicable research in their work tasks (Famutimi et al., 2015). Nurses also spend most time at work focusing on communication and hands-on tasks (Yen et al., 2018). It might be beneficial to consider more interdisciplinary work among NH physicians, pharmacists, and nurses to enhance each profession`s knowledge, skills, and competencies. This interdisciplinary collaborative approach may improve nurses’ pharmacology knowledge.

Previous studies assessing nurses’ knowledge of pharmacology show that the majority of participants seem to be senior nurses with long clinical experience. In Ndosi & Newell (2009) study, 95% of participating nurses had 9.3–16.2 years of work experience. This overrepresentation by senior nurses may be due to their professional confidence and willingness to participate (Ndosi & Newell, 2009). In contrast, our study had a different participant profile: 37.9% had 1–5 years work experience, 44.1% had 6–15 years work experience, and 17.9% had more than 16 years of work experience from NHs. Our results are in line with Markowitz et al. (1981) and Boggs et al. (1988), who found no significant difference in drug knowledge among nurses with different levels of work experience. The results thus indicate that clinical experience in itself may not enhance knowledge of pharmacology among nurses working in NHs. However, it is important to note that other studies report a positive association between years of clinical experience and knowledge of pharmacology and medication management (Ndosi & Newell, 2009; Simonsen et al., 2014). It has been suggested that in order to increase knowledge and competence, clinical experience should be combined with a specific learning program (Ives et al., 1996).

Our study shows no significant difference in the scores obtained by nurses with, and nurses without postgraduate education. None of the participants in our study had a postgraduation in pharmacology or medication management. This may indicate that graduate qualifications among participants are not necessarily relevant to pharmacology or medication management. An earlier study, however, has shown that nurses with postgraduate education achieved higher scores on pharmacology knowledge and medication management (Ndosi & Newell, 2009). A potential explanation for this, proposed by Ndosi & Newell (2009) is that senior nurses are more likely to have the opportunity for formal or self-directed learning (Ndosi & Newell, 2009) and are more engaged in medication-related activities, which make them more experienced in medication management (Ridge & While, 1995). Postgraduate nurses may be able to identify their educational needs before undertaking relevant modules for their clinical practice, such as pain management, applied pharmacology, or pathophysiology (Ndosi & Newell, 2009).

Safe and effective medication management is seen as a crucial nursing task and includes assessment, administration, monitoring, and evaluation of drug effects (Manias et al., 2004). Practicing nurses are expected to know the therapeutic uses, normal dosage, side effects, and precautions of the drugs they administer (Manias & Bullock, 2002). High-risk medications are defined as “medications associated with significant patient harm or death if they are misused or used in error” (Australian Commission on Safety and Quality in Health Care, 2020). Our questionnaire includes nine questions that we consider of special important clinical relevance among nurses working in NHs. Some of these questions are about high-risk medications. Lack of knowledge about these topics may have a clinically negative impact on the patient and compromise safe medication management (Stevenson et al., 2014). There is no standardized international list of high-risk medications that specifically applies to NH patients, but opioids and anticoagulants are often classified as high-risk medications (Sluggett et al., 2020). We set the cut score to eight of nine correct answers when evaluating he nurses’ knowledge of these drugs, because ensuring the appropriate use of high-risk medications is important to minimize the risk of medication-related harm in this vulnerable population (Sluggett et al., 2020) and traditionally in Norway, a faultless test in drug calculation during nursing education is required. The results show that only 18.6% of the nurses have adequate overall knowledge of high-risk medications. Furthermore, the results also show that nurses working in NHs had inadequate knowledge about opioids; 64.1% of nurses had adequate knowledge about the drugs. Opioids are often used in older patients for the management of moderate-to-severe pain (Davies, 2017). The use of these substances increases the risk of central nervous system depression, falls and fractures, and sedation and delirium (Davies, 2017). To minimize potential harm, it is crucial that nurses understand the pharmacology, side effects, and risks of opioids for the safe administration of these drugs (Telford, 2020). Our results show that 64.1% of the nurses had adequate knowledge about anticoagulants. These drugs are considered the first line of treatment for venous thromboembolism and they are also used for the treatment and prevention of blood clots (Gee, 2018). The risk of ischemic stroke increases significantly with age, making anticoagulants one of the medications most frequently prescribed in elderly patients as antithrombotic therapy (Lip et al., 2010; Robert-Ebadi et al., 2009; Singer et al., 2013). However, aging also increases the risk of anticoagulant-associated bleeding complications (Shah et al., 2019). Nurses need specific knowledge of pharmacology to understand the antithrombotic effect of the anticoagulants and the risk of bleeding. An earlier study has shown that anticoagulants are a drug class that frequently causes patients to visit the emergency department due to bleeding incidents (Shehab et al., 2016). Given that high-risk medication has only small margins of safety and that many of these medications are frequently administered by nurses, we find the lack of knowledge alarming, as this may lead to unsafe medication management. This is also highlighted in previous studies, which have found that two-thirds of hospital emergency admissions for adverse medication reactions in older patients were related to high-risk medications (Budnitz et al., 2011).

Generic substitution is common in hospitals and NHs (Tachi et al., 2018) and safe medication management requires knowledge of the prerequisites for using generic substitution. A study conducted by Hakonsen et al. (2010) in a Norwegian hospital has shown that nurses have inadequate knowledge about generic substitution. Of the participating nurses in this study, 42% experienced medication error as a result of generic substitution. These findings are corroborated by another study conducted in a Norwegian hospital, which shows that nurses lack essential knowledge about generic substitution (Johansen, 2019). Our study supports these findings. This question was answered by 198 of the 344 nurses, and only 33.5% knew the prerequisites for generic substitution. The main concern is that nurses are using the ATC register as a source of information for generic substitution instead of using the approved substitution list issued by The Norwegian Medical Products Agency (Johansen, 2019).

The results show that only 54.5% of the nurses can differentiate between agonists and antagonists. Knowledge of pharmacodynamics is crucial for understanding how drugs affect the body. Understanding the differences between agonist and antagonist actions allows the nurse to anticipate the expected actions of the medication and the patient response to teach patients how to prevent and manage medical conditions (Nolan et al., 2001).

Previous studies have shown that practicing nurses have insufficient knowledge of pharmacology and medication management (Grandell-Niemi et al., 2005; Manias, 2009; Manias & Bullock, 2002; Meechan et al., 2011; Ndosi & Newell, 2009). These findings are consistent with our results. This inadequate level of knowledge may be due to the inadequate teaching of pharmacology to undergraduate students and few post-registration pharmacology education opportunities (King, 2004; Latter et al., 2000; Morrison-Griffiths et al., 2002; Simonsen et al., 2014). A survey of lecturers in pharmacology reveals general dissatisfaction with the extent to which pharmacology is taught and a wish for the subject to be taught more (Latter et al., 2000). Furthermore, the nurses rated pharmacology teaching on their undergraduate program as insufficient (Latter et al., 2000). The nurses also reported a lack of confidence when speaking to patients about their medications (Latter et al., 2000). Some previously identified factors that may also explain the inadequate knowledge of pharmacology and medication management among nurses include few teaching hours in pharmacology in basic education (Khan & Hood, 2018), too much focus on correct drug calculation only (Khan & Hood, 2018), the low level of pharmacology knowledge among teachers, and an inadequate integration of pharmacology into clinical practice (Khan & Hood, 2018).

Nursing homes are highly collaborative environments, where nurses work closely with other healthcare professionals (Potrebny et al., 2022). However, nurses often work alone and have a high degree of professional responsibility (Svendsen et al., 2017). Nurses primarily care for elderly residents who often have chronic illnesses, cognitive impairments, and require long-term care. The work involves often long-term patient relationship. Besides understaffing, the challenging work tasks require training in geriatric care, dementia care, and palliative care to effectively meet the needs of their residents (Potrebny et al., 2022). Nurses need to update their pharmacological knowledge regularly to meet the demands of health care settings. Continuing professional development (CPD) programs are necessary to achieve this goal (Cleary-Holdforth & Leufer, 2013). Norway is a nonmandatory CPD country (Mlambo et al., 2021). In Norway, nurses frequently acquire knowledge through unstructured and informal workplace learning, such as discussing with colleagues, using the internet, or contacting experts in the field (Kyrkjebø et al., 2017).

In Norway, the time given for the teaching of pharmacology, pathology, clinical medicine, medical tests, and diagnostic procedures is generally around three months during the first year of a three-year undergraduate nursing program. Postgraduate pharmacology education opportunities do not exist (Fagerli, 2023). A survey of 262 nurses practicing in NHs in Norway revealed that more than 60% of the participants stated that there is a need for more knowledge of age-related physiological changes and pharmacology. Nearly all participants agreed that staff members responsible for medication management should take a mandatory medication management course, as self-directed learning rarely occurs (Wannebo & Sagmo, 2013).

## Strengths and Limitations

The focus of this study has been to examine the knowledge of pharmacology and medication management among nurses in NHs. Our results are from a multicenter study involving 24 NHs. Our study examines various aspects about nurses’ knowledge in pharmacology and medication management. Earlier studies were more concentrated on investigating specific topics such as medication errors in nursing practice (Brady et al., 2009; Furjanic et al., 2016) or the pharmacology of pain management (Furjanic et al., 2016), and many findings were derived from self-assessing tests (Qedan et al., 2022).

The present study has some limitations. The completion process of the questionnaire was not controlled. This may have made it possible for respondents to cooperate, refer to textbooks, or use the internet. Another limitation is the construction of the questionnaire was not validated. This instrument was developed specifically for this study. Several nurses were excluded from the main statistical analysis because they did not answer all the questions. This may indicate that the overall test scores were lower than our results show. We are also aware that the use of multiple-choice questions with one in four answers being correct, inherently allows for a 25% correct answer rate by chance. This requires us to raise the limit for the minimum requirement accordingly.

The Covid-19 pandemic and the consequences thereof may have made it more demanding to recruit nurses. Most of the restrictions in Norway were downscaled in the period during the data collection. However, recent research has shown that the COVID-19 pandemic had a significant impact on nurses’ performance, knowledge, and ability to provide care for the patients. The pandemic required increased workloads which resulted in stress and burnout. Many nurses experienced anxiety and depression due to the high stress in work environment (Blanchard et al., 2022; de Vos et al., 2024). During the pandemic, the nurses were busy with increased training and upskilling efforts to stay updated about the latest treatment protocols (de Vos et al., 2024). These work conditions may have negatively influenced the nurses’ s performance when carrying out the pharma-1 test.

## Implications for Practice

The inadequate knowledge in pharmacology and medication management is worrying since adequate knowledge in pharmacology and medication management is essential for reducing medication errors that are potentially harmful for the patient. The findings suggest that a higher level of focus is important to pharmacology courses in undergraduate nursing education and that more effort should be made to improve practicing nurses’ knowledge of pharmacology through purposeful learning programs such as collaborative pharmacology courses.

## Conclusions

Nurses working with vulnerable patients in NHs have a vital role to play in providing safe medication management. The present study shows that nurses working in NHs in Norway have insufficient knowledge of pharmacology and medication management. Age, work experience, and postgraduate education do not influence the nurses’ knowledge. Nurses have a better knowledge in medication management when compared to general or clinical pharmacology. They also have poor knowledge of specific groups of high-risk drugs such as anticoagulants and opioids, generic drugs, and pharmacological concepts such as the difference between agonists and antagonists.

## Supplemental Material

sj-docx-1-son-10.1177_23779608241303482 - Supplemental material for Pharmacology Knowledge Among Nurses Working in Nursing Homes in Norway: A Cross-Sectional Study Supplemental material, sj-docx-1-son-10.1177_23779608241303482 for Pharmacology Knowledge Among Nurses Working in Nursing Homes in Norway: A Cross-Sectional Study by Siv Eriksen Taasen, Fred-Ivan Kvam, Kjersti Marie Blytt and El Houcine Messaoudi in SAGE Open Nursing

sj-docx-2-son-10.1177_23779608241303482 - Supplemental material for Pharmacology Knowledge Among Nurses Working in Nursing Homes in Norway: A Cross-Sectional Study Supplemental material, sj-docx-2-son-10.1177_23779608241303482 for Pharmacology Knowledge Among Nurses Working in Nursing Homes in Norway: A Cross-Sectional Study by Siv Eriksen Taasen, Fred-Ivan Kvam, Kjersti Marie Blytt and El Houcine Messaoudi in SAGE Open Nursing
