# Supplementary material for: Pharmacology Knowledge Among Nurses Working in Nursing Homes in Norway: A Cross-Sectional Study
Source: SAGE Open Nurs. 2024 Dec 5;10:23779608241303482. doi: 10.1177/23779608241303482 (PMC11622299; doi:10.1177/23779608241303482)
Supplement: sj-docx-2-son-10.1177_23779608241303482 - Supplemental material for Pharmacology Knowledge Among Nurses Working in Nursing Homes in Norway: A Cross-Sectional Study [file sj-docx-2-son-10.1177_23779608241303482.docx]

**Pharma-test 1**

**1.0 Demographic data**

| 1.1 Male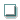Female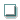 |
| --- |
|  |
| 1.2 Age: 20-25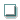26-35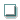36-45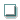46-55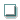56-65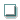66-70 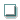Years |
|  |

| 1.3 Optional question: Here were the nursing homes listed. |
| --- |
|  |
| 1.4 What type of department do you work in?  Long-term care 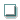 Short-term care 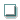 |
|  |

| 1.5 Number of years since completion of basic education in nursing: |
| --- |
| 1-5 years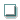6-10 years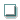11-15 years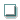More than 16 years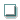 |
| 1.6 Number of years’ work experience as a nurse: |
| 1-5 years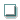6-10 years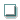11-15 years 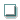More than 16 years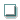 |

| 1.7 Education: |
| --- |
| Registered nurse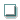 Dementia approx. 10 credits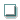Geriatrics 60 credits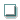  Palliative care 30-60 credits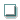Cancer nursing 60 credits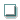Psychiatry 60 credits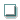  Relevant master’s degree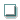Organisation and management 60 credits.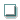Emergency room nursing 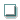  Other relevant education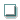 |
| 1.8 Position percentage? |
| \| 100% \| 80% \| 70% \| 60% \| 50% \| 40% \| 30% \| 20% \| 10% \| Call attendant with variable working hours \| \| --- \| --- \| --- \| --- \| --- \| --- \| --- \| --- \| --- \| --- \| \| 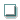 \| 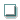 \| 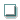 \| 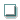 \| 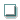 \| 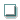 \| 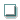 \| 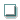 \| 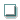 \| 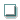 \| |

**2.0 General pharmacology**  
Each question has one correct answer.

| 2.1 Many patients have trouble swallowing tablets. Some nurses therefore choose to crush them.  Which tablet can be crushed? |
| --- |
| a) Isoptin retard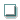b) Albyl-E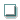c) Duroferon depot tablet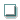 d) Apocillin tablet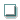e) Kaleorid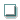 |
| 2.2 Many elderly patients use drugs with anticholinergic side effects. One possible anticholinergic  side effect is: |
| a) Dry mouth 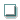  b) Dehydration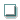  c) Weight reduction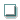  d) Hypertension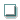 |
| 2.3 You are about to hand out medication to a patient in a nursing home when you discover that you  are running short of a drug on the patient’s drug list. What are the prerequisites for using  a generic drug instead? |
| a) The state’s pharmaceuticals agency has approved the drugs as interchangeable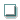  b) The drugs contain the same active ingredient in an equal dosage and concentration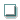  c) The drugs have the same pharmaceutical form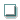  d) The drugs are listed with the same ATC code in the Norwegian Pharmaceutical Product Compendium |
| (Felleskatalogen) 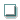  2.4 Interaction is common when two or more drugs are given simultaneously.  Why should you be careful when combining the administration of NSAIDs and ACE inhibitors to  elderly patients? |
| a) NSAIDs inhibit intake of ACE inhibitors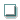  b) The combination of these two drugs affects renal filtration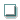  c) The combination of these two drugs increases the risk of gastrointestinal bleeding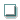  d) The combination of these two drugs can prolong the QT interval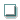 |

| 2.5 Which is the correct statement about therapeutic range? |
| --- |
| a) Penicillin has a narrow therapeutic range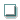  b) Marevan (warfarin) has a narrow therapeutic range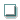  c) Lithium has a wide therapeutic range 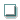  d) Cytostatics have a wide therapeutic range 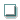 |
| 2.6 Which of the following drugs may have antidopaminergic side effects? |
| a) Antiepileptic drugs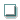  b) Antipsychotics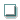  c) Benzodiazepines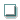  d) Antidiabetics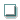 |
| 2.7 Cardiovascular drugs: Many elderly patients suffer from hypertension. How is hypertension treated? |
| a) Statins and lifestyle changes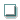  b) Acetylsalicylic acid and lifestyle changes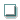  c) Antihypertensives and lifestyle changes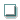  d) Statins and beta-2 stimulators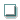 |
| 2.8 A patient is using opioids (OxyNorm) as a treatment for strong pain.  It has been decided that the treatment will change from oral to parenteral administration.  This implies that the patient will have: |
| a) A higher dose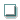  b) The same dose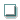  c) A lower dose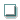  d) Another opioid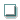 |
| 2.9 Some drugs influence the liver’s ability to break down other drugs. These drugs are called enzyme  inducers or enzyme inhibitors. Which of the following statements is correct?' |
| a) Enzyme inhibitors may lead to the reduced effect of other drugs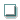  b) An enzyme inducer may lead to the increased effect of other drugs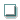  c) The enzyme inducer carbamazepine (an anticonvulsant) reduces the effect of warfarin (Marevan)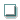  d) Natural remedies such as St John’s wort have a small influence on liver metabolism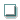 |

**3.0 Medication management**

| 3.1 What is the right way to take a Remeron S tablet? |
| --- |
| a) Dissolved in water before intake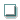  b) Placed on the tongue, where it quickly dissolves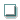  c) Divided in two before intake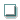  d) Placed under the tongue where it quickly gets absorbed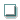 |

| 3.2) Albyl-E is used to prevent the formation of blood clots. What is the mechanism of action of Albyl-E?  a) Albyl-E inhibits coagulation factors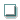  b) Albyl-E stimulates coagulation factors 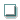  c) Albyl-E inhibits the function of thrombocytes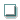  d) Albyl-E increases INR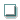  3.3) Which statement about the use of medicinal patches is correct? |
| --- |
| a) Medicinal patches can be used if a steady supply of a drug is desired over time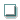  b) Medicinal patches give a quick effect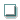  c) Medicinal patches work best if you put them in the same place every time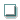  d) Medicinal patches have fewer side effects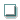 |
|  |
| 3.4) Why should certain tablets not be crushed? |
| a) In powder form, it is difficult to administer the full dose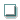  b) The active substance is destroyed when the tablets are crushed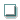  c) The tablets’ long-term or protective effects are destroyed when they are crushed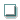  d) The crushing of tablets always leads to increased absorption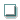 |

| 3.5) After inhaling a dose of an asthma drug containing steroids, it is important to: |
| --- |
| a) Lie down a little while to let the drugs work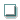  b) Clean the inhaler with water to remove drug residues and saliva 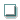  c) Rinse the mouth well and spit out any residues of the drug 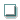  d) Eat food to reduce side effects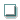 |
| 3.6) Which organ is responsible for most of the breakdown of drugs? |
| a) Stomach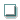  b) Pancreas 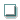  c) Kidneys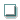  d) Liver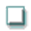  **4.0 Medication for cardiovascular disease** |
| 4.1) Which statement about agonists and antagonists is correct? |
| a) An antagonist is a drug that stimulates a receptor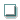  b) An antagonist is a drug that blocks a receptor  c) A partial agonist is a drug that provides only partial stimulation of a receptor  d) A drug that enhances the effect of an agonist is called a partial antagonist |
| 4.2) Marevan (warfarin) is used to prevent blood clots. Choose the correct statement about Marevan: |
| a) Alcohol inhibits the effect of Marevan  b) Foods containing vitamin K lower INR, thus reducing the effect of warfarin  c) Marevan must be administered by injection  d) Marevan increases the formation of vitamin K-dependent coagulation factors |
|  |
| 4.3) Many patients use loop diuretics/drugs such as furosemide against hypertension and/or  heart failure. What is a common side effect of using furosemide? |
| a) High doses of ACE inhibitors may induce hypotension  b) Long-term use may induce hypernatremia  c) Long-term use may induce hyperkalemia  d) Furosemide may be used for the treatment of gout |
|  |

**5.0 Medicines that act on the respiratory organs**

| 5.1) Asthma attacks can be triggered by: |
| --- |
| a) The use of NSAIDs  b) Hypotension  c) The use of drugs against chronic obstructive pulmonary disease (COPD)  d) Anemia |
| 5.2) The most common drug treatment for asthma is: |
| a) Beta-2 antagonists and NSAIDs  b) Beta-2 agonists and NSAIDs  c) Beta-2 antagonists and glucocorticoids  d) Beta-2 agonists and glucocorticoids |
| 5.3 Atrovent (ipratropiumbromide) is an anticholinergic drug used in the treatment of chronic  obstructive pulmonary disease (COPD) and asthma. Choose the correct statement about Atrovent: |
| a) Increases oxygen uptake in the lungs  b) Increases intracellular Ca^2+^ in the smooth muscles of the bronchi  c) Dilates the bronchi  d) Acts as an anti-inflammatory |

**6.0 Medicines that affect the stomach and intestinal system**

| 6.1) Several drugs can cause constipation. Which medicines can cause constipation as  a side effect? |
| --- |
| a) Opioids and iron tablets  b) Opioids and glucocorticoids  c) Opioids and antibiotics  d) Opioids and NSAIDs |
| 6.2) For oral iron therapy, which of the following treatment combinations is recommended? |
| a) Iron tablets administered with milk  b) Iron tablets administered with antacids  c) Iron tablets administered with vitamin K tablets  d) Iron tablets administered with vitamin C tablets |
| 6.3) How do proton pump inhibitors work? |
| a) They neutralise acidic gastric content  b) They increase muscle tone in the gastroesophageal sphincter  c) They mechanically prevent reflux  d) They reduce the production of acid in the stomach |
| 6.4) Treatment of diabetes: Metformin (Glucophage) is an oral antidiabetic drug.  Which patient group should avoid the use of metformin? |
| a) Patients with renal failure  b) Obese patients  c) Patients using sedatives  d) Patients using insulin |

**7.0 Painkillers (analgesics)**

| 7.1) Paracetamol has both an analgesic and an antipyretic effect. What is the half-life of paracetamol? |
| --- |
| a) Approx. 5 minutes b) Approx. 30 minutes c) Approx. 1 hour d) Approx. 2 hours |
| 7.2) Below are four claims about various painkillers and their side effects. Which one is correct? |
| a) NSAIDs work by stimulating the enzyme cyclooxygenase  b) One of the side effects of NSAIDs is stomach ulcers  c) Opioids (morphine) stimulate respiration by stimulating opioid receptors  d) Opioids (morphine) act as pain relievers by blocking opioid receptors (µ-receptors) in the CNS |
|  |

| 7.3) Hypnotics: The drugs most frequently used for sleep disorders are: |
| --- |
| a) Benzodiazepines and benzodiazepine-like hypnotics  b) Benzodiazepines and antihistamines  c) Benzodiazepines and antipsychotics  d) Benzodiazepines and melatonin |
| 7.4) Choose the correct statement about hypnotics: |
| a) Truxal (chlorprothixene) has a short duration of action  b) Melatonin has a very long duration of action  c) Zopiclone (zoplikon) has a short duration of action  d) Stilnoct (zolpidem) has a very long duration of action |

**8.0 Musculoskeletal system**

| 8.1) Some patients use drugs to prevent the development of osteoporosis. Choose the correct  statement about the treatment of osteoporosis: |
| --- |
| a) Tablets with bisphosphonates (Fosamax, Alendronate) should be taken with milk  to ensure the supply of calcium  b) Tablets with bisphosphonates should be swallowed with plenty of water while one is sitting  c) Patients should take calcium tablets (Calcigran) at the same time as bisphosphonates  d) It is common to give vitamin A at the same time as bisphosphonates |
| 8.2 Medicines that act on the central nervous system (CNS): The patient has symptoms of  depression and starts with an antidepressant. How long do you expect it to take until  the patient experiences the full effect of the antidepressant drug? |
| a) 1-2 weeks  b) 2-4 weeks  c) 4-6 weeks  d) It depends on the type of antidepressant the patient is using |

**9.0 Palliation, emergency medicine and antibiotics**

| 9.1) One of your patients at the nursing home is in the final phase of life and has been  prescribed medications for use in the final phase of life. The patient is troubled with heavy  breathing (dyspnea). Which drug would you suggest giving and at what dosage? |
| --- |
| a) Morphine 1.0-2.5 mg s.c.  b) Midazolam 2.0-2.5 mg s.c.  c) Haloperidol 0.5-2.5 mg s.c.  d) Glycopyrrolate (Robinul) 0.2 mg s.c. |
| 9.2) Emergency medicine: You give a patient an intravenous injection of penicillin.  After a few minutes, the patient says he feels a bit “strange”. He scratches his palms.  He feels dizzy and has difficulty breathing. You stop the injection and rush for the  following drug, which you administer before contacting a doctor: |
| a) Atropine 1 ml/mg i.v.  b) Solu-Cortef 100 mg (glucocorticoids) i.v  c) Adrenalin 1 mg/ml – 0.5 ml i.m  d) Phenergan 25 mg tablet |
|  |
| 9.3) Acute pulmonary edema is treated in a nursing home with which of the following: |
| a) Morphine, furosemide and glycerol trinitrate  b) Morphine, furosemide and beta stimulators  c) Morphine, corticosteroids and NSAIDs  d) Morphine, calcium antagonist and diuretics |
| 9.4) Antibiotics: What is the usual indication for starting with Penomax (amidinopenicillin)? |
| a) A urinary tract infection  b) Pneumonia  c) Erysipelas  d) Smallpox |

|  |
| --- |
|  |
